# Author Correction: Sb_2_S_3_-templated synthesis of sulfur-doped Sb-N-C with hierarchical architecture and high metal loading for H_2_O_2_ electrosynthesis

**DOI:** 10.1038/s41467-024-55733-6

**Published:** 2025-01-09

**Authors:** Minmin Yan, Zengxi Wei, Zhichao Gong, Bernt Johannessen, Gonglan Ye, Guanchao He, Jingjing Liu, Shuangliang Zhao, Chunyu Cui, Huilong Fei

**Affiliations:** 1 State Key Laboratory for Chemo/Biosensing and Chemometrics, and College of Chemistry and Chemical Engineering, Hunan University, Changsha, 410082 China; 2 Guangxi Key Laboratory of Petrochemical Resource Processing and Process Intensification Technology and School of Chemistry and Chemical Engineering, Guangxi University, Nanning, 530004 China; 3 Australian Synchrotron, ANSTO, Clayton, VIC 3168 Australia; 4 Advanced Catalytic Engineering Research Center of the Ministry of Education, Hunan University, Changsha, 410082 China

**Keywords:** Electrocatalysis, Electrocatalysis, Electrocatalysis

**Correction to:** *Nature Communications* 10.1038/s41467-023-36078-y, published online 23 January 2023

The original version of this Article contained a typographical error in the affiliation of the co-author, Bernt Johannessen. “Australian Synchrotron, Clayton, Victoria 3168, Australia” should have read “Australian Synchrotron, ANSTO, Clayton VIC 3168, Australia”. The corresponding affiliation in author information has been updated in both the PDF and HTML versions of the Article.

