# Supplementary material for: Sb2S3-templated synthesis of sulfur-doped Sb-N-C with hierarchical architecture and high metal loading for H2O2 electrosynthesis
Source: Nat Commun. 2023 Jan 23;14:368. doi: 10.1038/s41467-023-36078-y (PMC9871021; doi:10.1038/s41467-023-36078-y)
Supplement: Supplementary file 1 — Supplementary Information [file 41467_2023_36078_MOESM1_ESM.pdf]

Supplementary Materials for

Sb_2S_3-templated synthesis of sulfur-doped Sb-N-C catalyst with hierarchical architecture and high metal loading for H_2O_2 electrosynthesis

Minmin Yan[1#], Zengxi Wei[2#], Zhichao Gong[1], Bernt Johannessen,[3] Gonglan Ye[1,], Guanchao He[1], Jingjing Liu[1], Shuangliang Zhao[2,*], Chunyu Cui[1,*], Huilong Fei[1,4,*]*

[1]State Key Laboratory for Chemo/Biosensing and Chemometrics, and College of Chemistry and Chemical Engineering, Hunan University, Changsha 410082, China; [2]Guangxi Key Laboratory of Petrochemical Resource Processing and Process Intensification Technology and School of Chemistry and Chemical Engineering, Guangxi University, Nanning 530004, China; [3]Australian Synchrotron, Clayton, Victoria 3168, Australia; [4]Advanced Catalytic Engineering Research Center of the Ministry of Education, Hunan University, Changsha 410082, China.

*Correspondence to: szhao@gxu.edu.cn; cycui@hnu.edu.cn; glye@hnu.edu.cn; hlfei@hnu.edu.cn

#These authors contributed equally to this work

Contents:

Supplementary Figures 1 to 32

Supplementary Tables 1 to 3

Supplementary Figures

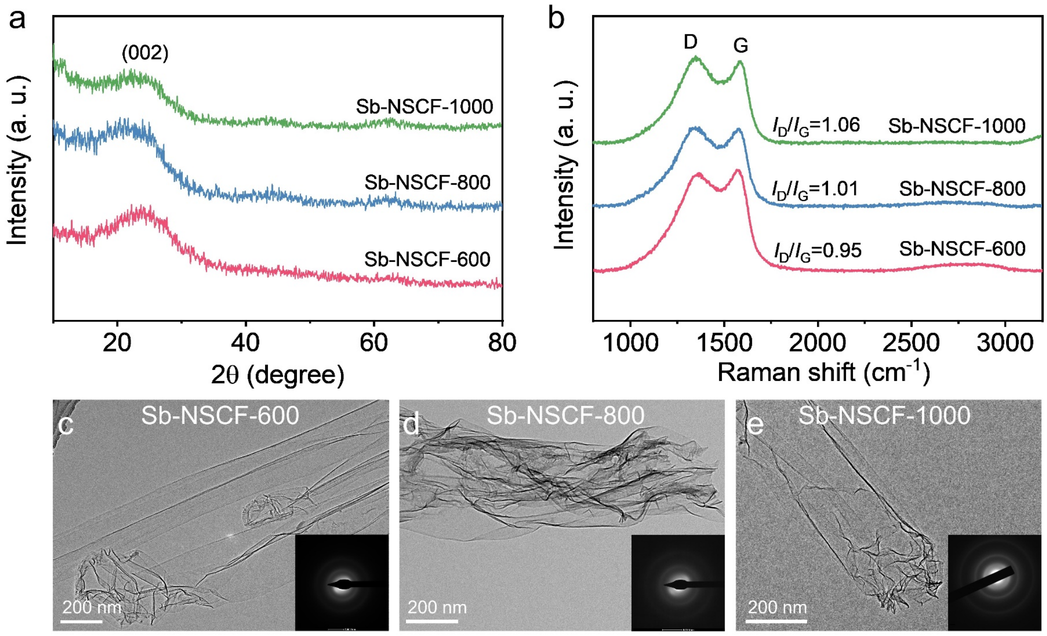

Supplementary Figure 1. a, XRD patterns of Sb-NSCF-600, Sb-NSCF-800 and Sb-NSCF-1000. **b**, Raman spectra of Sb-NSCF-600, Sb-NSCF-800 and Sb-NSCF-1000. **c**–**e**, TEM images of Sb-NSCF-600, Sb-NSCF-800 and Sb-NSCF-1000. The inset figures in **c**–**e** show the SAED of the corresponding sample. To study the influence of temperature on the degree of graphitization and electrocatalytic performances, the synthesis temperature was varied to prepare different samples, which were denoted as Sb-NSCF-600, Sb-NSCF-800 and Sb-NSCF-1000 with the number denoting the synthesis temperature. The results show that all samples have similar degree of graphitization and are highly defective, as evidenced by the broad XRD peak at ~ 23.6° corresponding to the (002) plane of carbon, the large I_D/I_G ratio in the Raman spectra and the diffused halo ring of the selected-area electron diffraction (SAED).

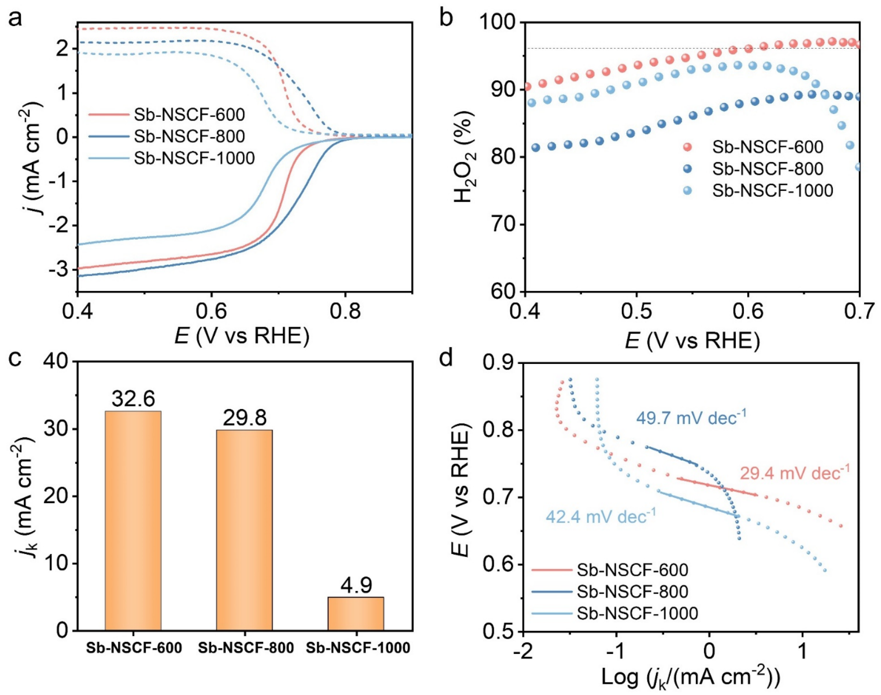

Supplementary Figure 2. a, Electrochemical oxygen reduction polarization curves (solid lines) at a rotation of 1600 rpm and simultaneous H_2O_2 detection currents on the ring electrode (dashed lines) for Sb-NSCF-600, Sb-NSCF-800 and Sb-NSCF-1000. **b**, Calculated H_2O_2 selectivity (%) on Sb-NSCF-600, Sb-NSCF-800 and Sb-NSCF-1000. **c**, Comparison of H_2O_2 kinetic current density (j_k) at 0.65 V. **d**, Comparison of Tafel slopes. The results show that the catalytic performances are sensitive to the synthesis temperature. Specifically, Sb-NSCF-600, Sb-NSCF-800 and Sb-NSCF-1000 have maximized H_2O_2 selectivity of 97.2%, 89.3% and 93.6%, respectively. Moreover, Sb-NSCF-600 possesses the highest kinetic current density (j_k) of 32.6 mA cm^{-2} at 0.65 V, exceeding those of Sb-NSCF-800 (29.8 mA cm^{-2}) and Sb-NSCF-1000 (4.9 mA cm^{-2}). Furthermore, the analysis of Tafel plots reveals that Sb-NSCF-600 presents a Tafel slope of 29.4 mV dec^{-1}, smaller than those of Sb-NSCF-800 (49.7 mV dec^{-1}) and Sb-NSCF-1000 (42.4 mV dec^{-1}). Therefore, Sb-NSCF-600 was identified as the optimal sample.

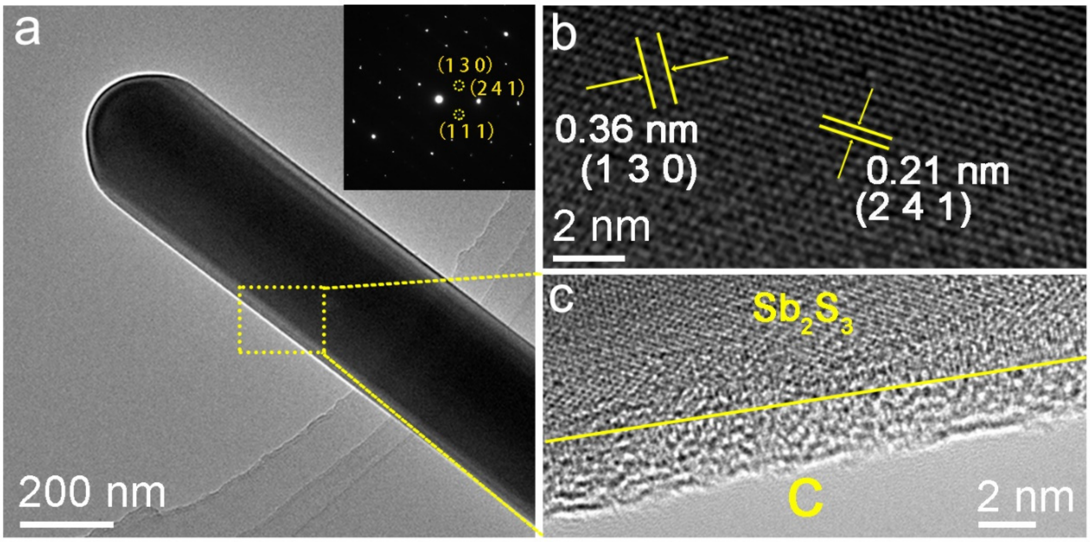

Supplementary Figure 3. a–c, TEM images of Sb_2S_3-NSC at different magnifications. Inset of (**a**) shows the SAED of Sb_2S_3-NSC. The TEM images in (**a**, **c**) reveal a core-shelled structure with Sb_2S_3 nanorods encapsulated in carbon sheath, the thickness of which is ~ 5.7 nm. The lattice spacings in (**b**) was evaluated to be approximately 0.36 nm and 0.21 nm, corresponding to the (130) and (241) planes of Sb_2S_3 (PDF#42-1393), respectively, indicting the crystalline structure of Sb_2S_3 at the core.

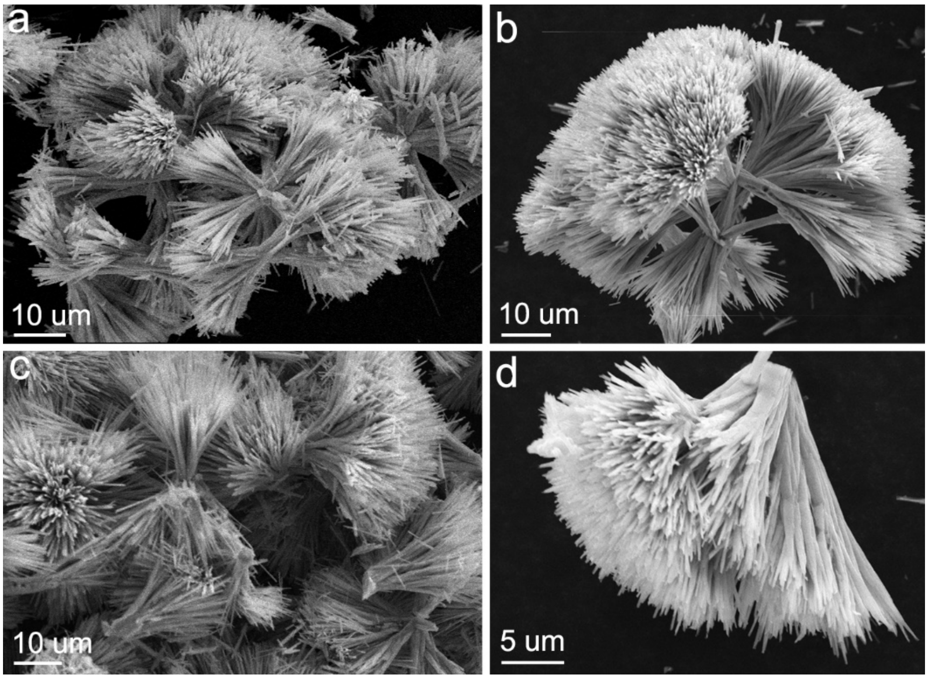

Supplementary Figure 4. a–d, SEM images of Sb_2S_3-NSC at different magnifications. The results show that the flower-like architecture assembled with carbon-coated Sb_2S_3 nanofibers.

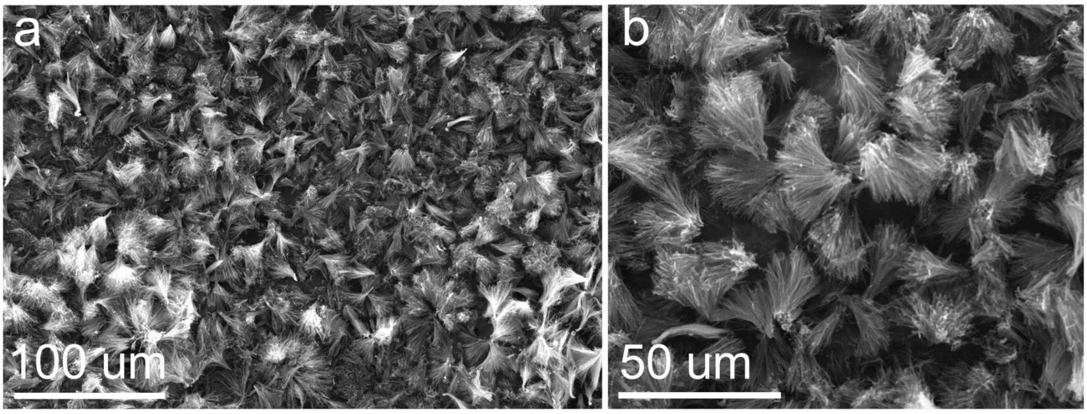

Supplementary Figure 5. SEM images of Sb-NSCF at low (**a**) and high (**b**) magnifications. The morphology of the flower-like structures is consistent throughout the sample.

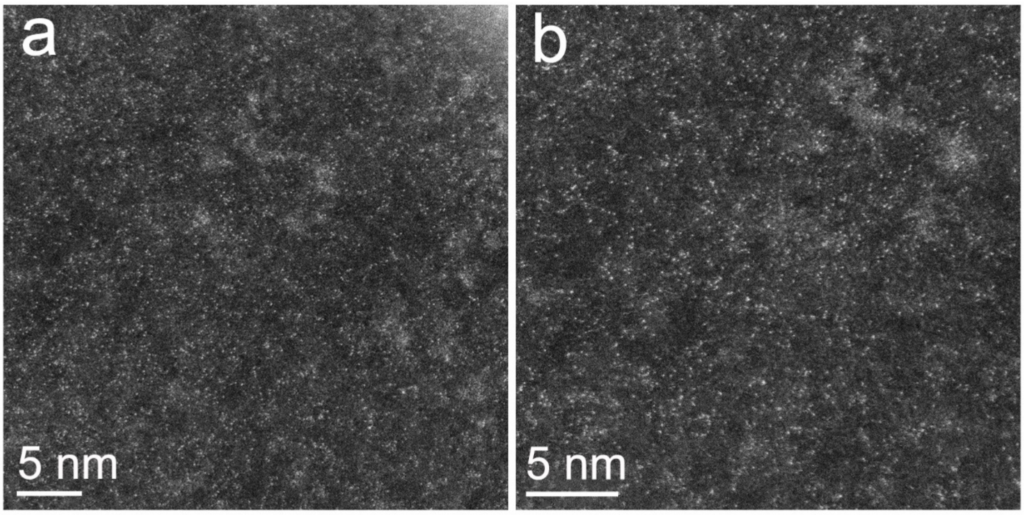

Supplementary Figure 6. a, **b**, HAADF-STEM images of Sb-NSCF. The results show that the Sb atoms are densely and uniformly distributed in the carbon matrix.

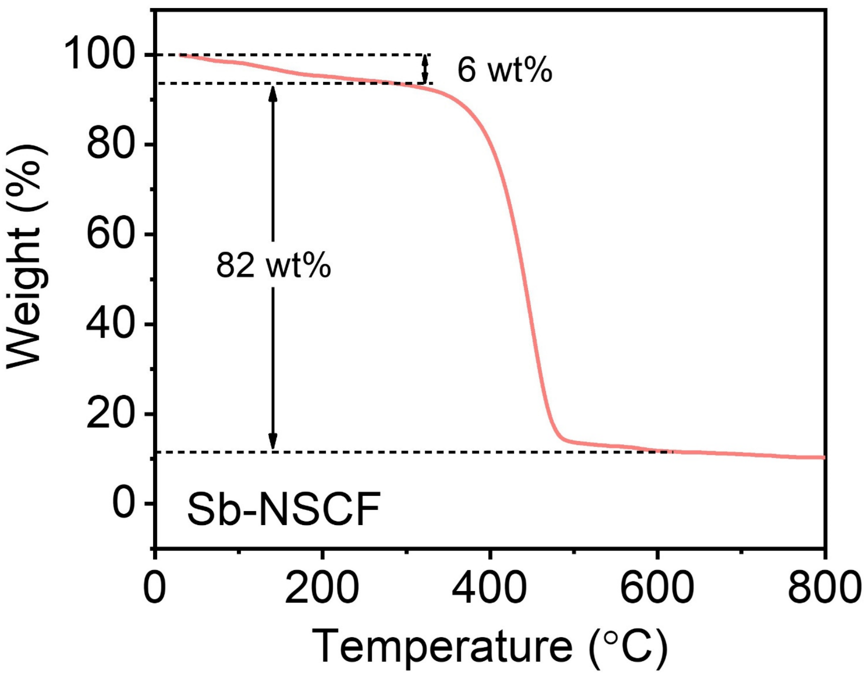

Supplementary Figure 7. TG analysis of Sb-NSCF performed in air atmosphere. The Sb content in Sb-NSCF was estimated to be 10.1 wt% from the equation below, where Sb_2O_4 is the oxidation product of Sb[1]. The 6 wt% loss at 30 – 250 °C is attributed to the evaporation of absorbed water in Sb-NSCF. The weight loss at 250 – 600 °C is attributed to the combustion of carbon[2].

$$\text{Sb (\%)} = \frac{2 \times \text{molecular weight of Sb}}{\text{molecular weight of } Sb_2O_4} \times \frac{\text{weight of } Sb_2O_4}{\text{weight of Sb-NSCF}} \times 100\%$$

$$= \frac{243.5}{307.5} \times \frac{1\text{-}0.06\text{-}0.82}{1\text{-}0.06} \times 100\% = 10.1\ \% \quad (1)$$

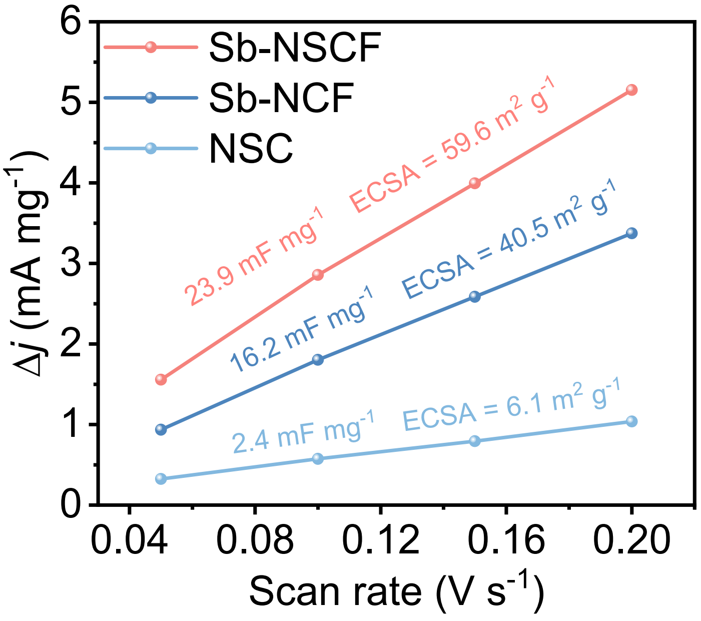

Supplementary Figure 8. The electrochemical double-layer capacitance and ECSA of different samples. The ECSA of a catalyst sample is calculated from the mass-normalized double-layer capacitance according to equation below:

$$\mathrm{ECSA} = \frac{C_{\mathrm{dl}}}{C_{\mathrm{s}}} \quad (2)$$

where C_{dl} is the measured double-layer capacitance. The C_{s} is the specific capacitance of an atomically smooth planar surface. For our estimates of surface area, we used a general specific capacitance of $C_{\mathrm{s}} = 0.040\ \mathrm{mF\ cm^{-2}}$ based on typical reported values[3].

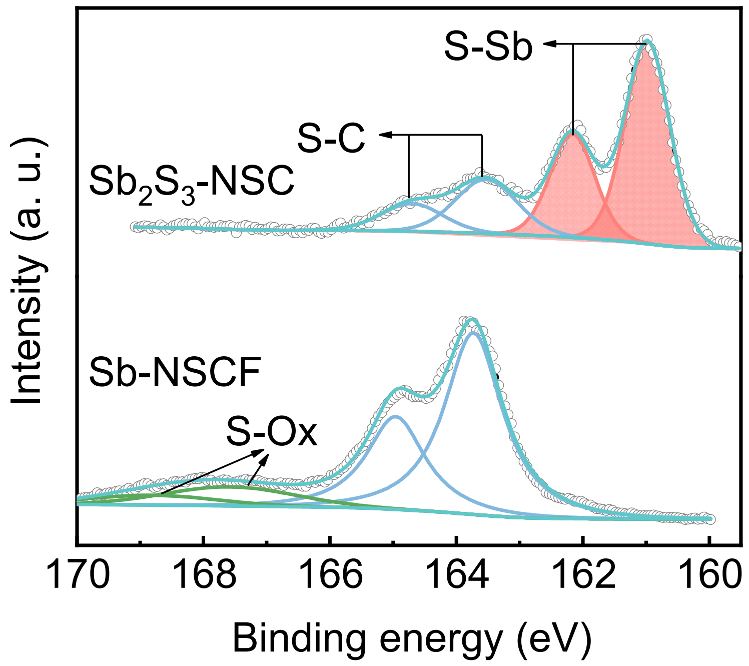

Supplementary Figure 9. The high-resolution S 2*p* spectrum of Sb_2S_3-NSC and Sb-NSCF. The results show that the peak for the S-Sb bonding in Sb_2S_3-NSC was at 161.0 eV and 162.2 eV, which disappeared in Sb-NSCF, suggesting the absence of Sb_2S_3 particles or clusters in Sb-NSCF.

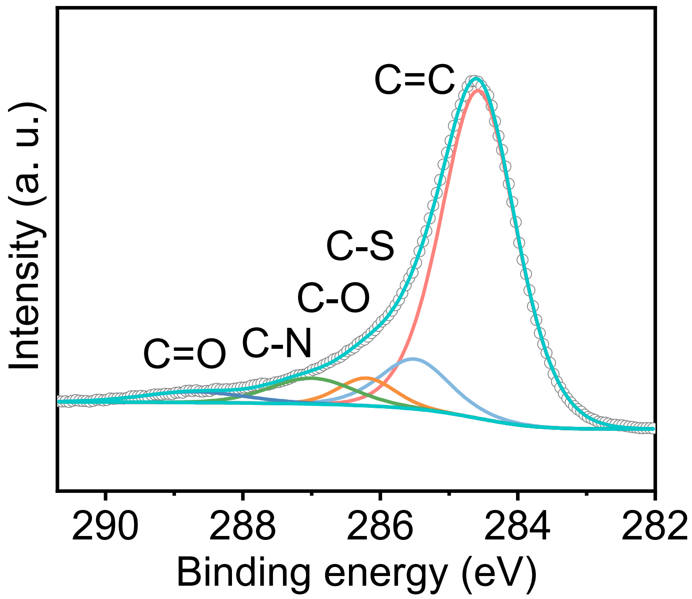

Supplementary Figure 10. The high-resolution C 1*s* spectrum of Sb-NSCF. The high-resolution C 1*s* spectrum can be deconvoluted into main peaks located at 284.6 eV, 285.5 eV, 286.2 eV, 287.0 eV, and 288.7 eV, corresponding to C=C, C-S, C-O, C-N, and C=O, respectively, validating that the non-metal elements (S and N) were successfully doped into the hollow carbon fibers[4].

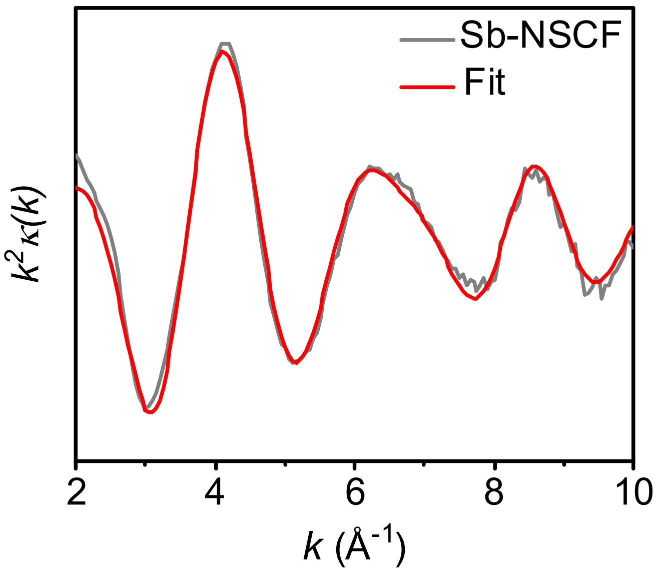

Supplementary Figure 11. FT-EXAFS fitting curves of Sb-NSCF at *k* space.

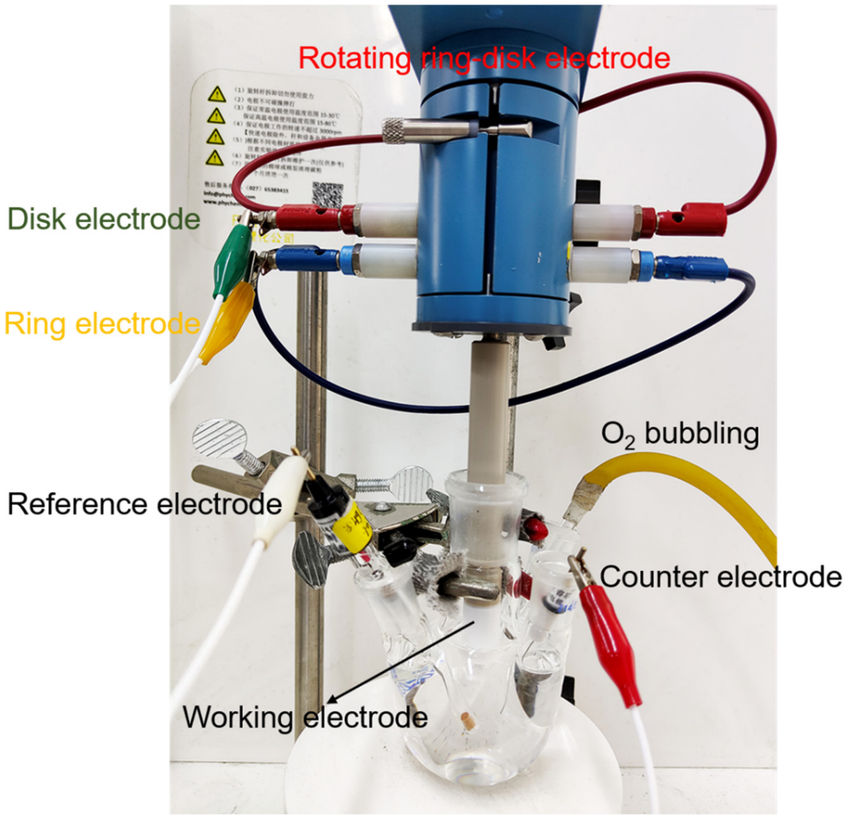

Supplementary Figure 12. Digital photograph of a typical rotating ring-disk electrode (RRDE) setup for the electrochemical $2e^-$ ORR measurements.

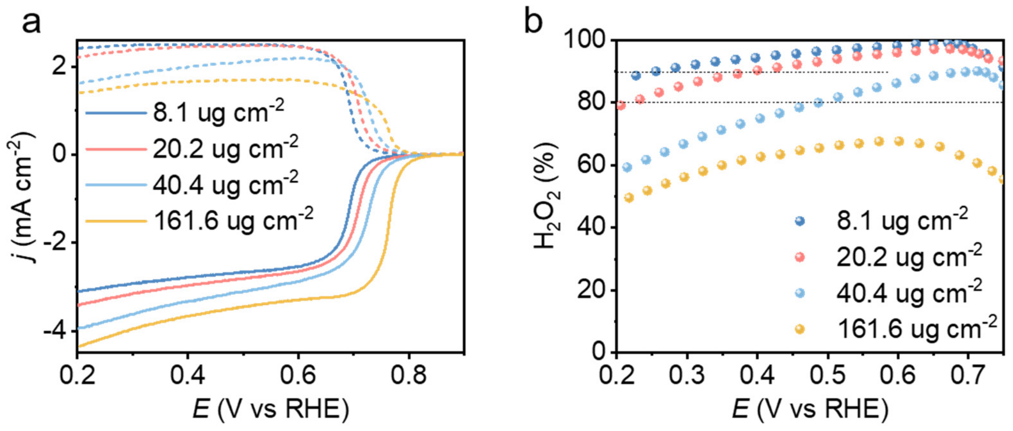

Supplementary Figure 13. Optimization of the loading amounts of the Sb-NSCF catalysts on the disk electrode. **a**, Electrochemical oxygen reduction polarization curves (solid lines) at a rotation of 1600 rpm and simultaneous H_2O_2 detection currents on the ring electrode (dashed lines) for Sb-NSCF with different loadings on the disk electrode. **b**, Calculated H_2O_2 selectivity (%) on Sb-NSCF with different loadings.

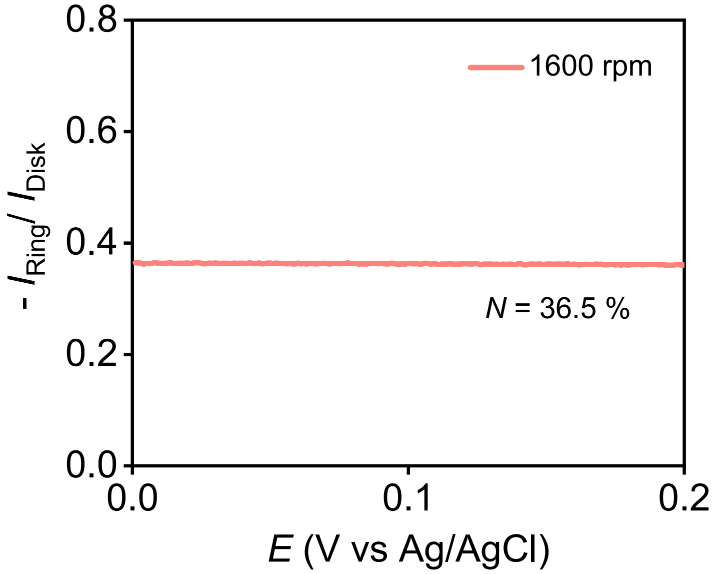

Supplementary Figure 14. Calibration of the collection efficiency (*N*) of RRDE electrode. The collection efficiency (*N*) of the RRDE was calculated from LSV polarization curves scanned in N_2-saturated 0.1 M KCl electrolyte containing 10 mM potassium ferricyanide ($K_3Fe(CN)_6$) using the equation: $N = -I_{Ring} / I_{Disk}$, where I_{Ring} is the current on Pt ring and I_{Disk} is the current on glassy carbon disk. The collection efficiency of the used RRDE was determined to be 0.365.

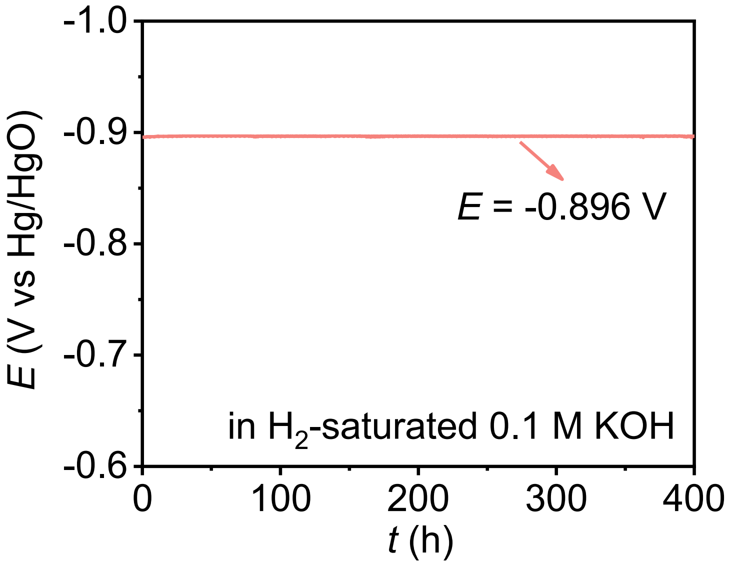

Supplementary Figure 15. Calibration of Hg/HgO reference electrode with respect to reversible hydrogen electrode (RHE) in H_2-saturated 0.1 M KOH. $E_{(RHE)} = E_{(Hg/HgO)} + 0.896$ V.

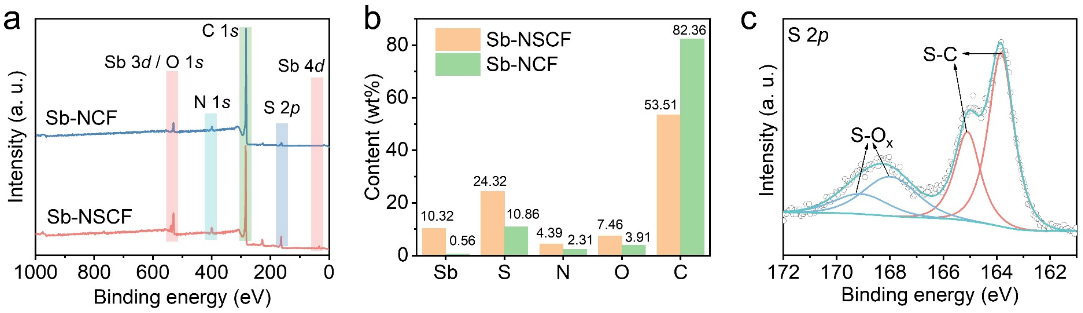

Supplementary Figure 16. a, XPS survey spectrum of Sb-NSCF and Sb-NCF. **b**, Chart showing the content of Sb, S, N, O, and C elements in Sb-NSCF and Sb-NCF measured by XPS. The results show that the contents of Sb and S elements are greatly decreased in Sb-NCF in comparison to Sb-NSCF. The content of S is significantly decreased from 24.32 wt% in Sb-NSCF to 10.86 wt% in Sb-NCF. **c**, The high-resolution S 2*p* spectrum of Sb-NCF. The results show that the most dominant peaks are originated from C-S bonds (163.8 and 165.1 eV), while there are also two minor peaks corresponding to S-O_x bonds (168.0 and 169.1 eV).

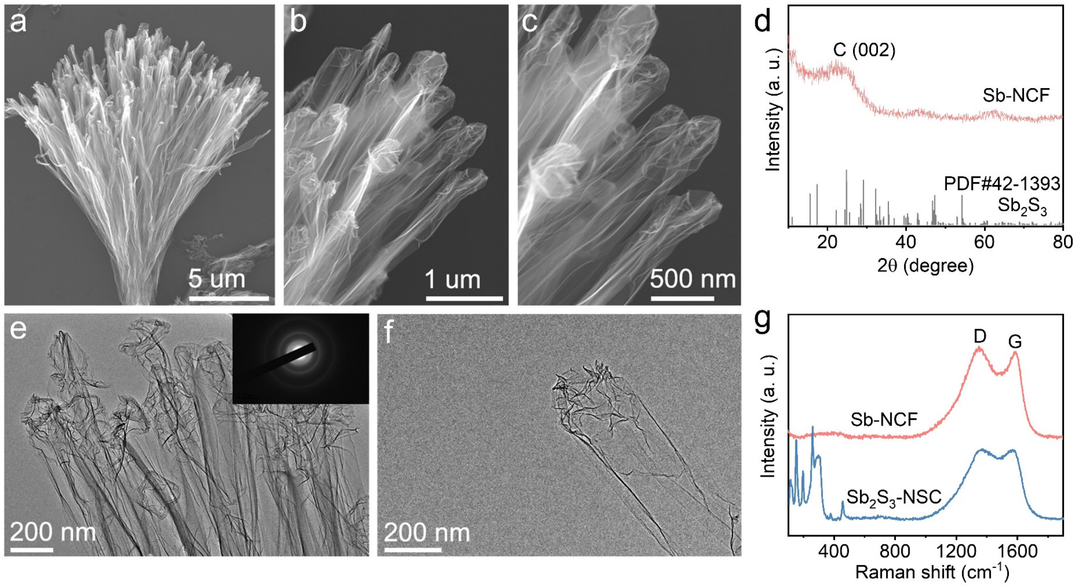

Supplementary Figure 17. a–c, SEM images of Sb-NCF at different magnifications, which shows the flower-like hollow carbon fiber morphology, similar to Sb-NSCF. **d**, XRD patterns of Sb-NCF and the Sb_2S_3 standard. The broad C (002) peak suggests the amorphous feature of carbon in Sb-NCF. **e**, **f**, TEM images of Sb-NCF at different magnifications. The inset of (**e**) shows the SAED of Sb-NCF, which indicates the amorphous structure of carbon in Sb-NCF, consistent to the XRD results. **g**, Raman spectra of Sb-NCF and Sb_2S_3-NSC. No signal of Sb_2S_3 was detected, indicating the complete removal of Sb_2S_3. The high D-to-G band ratio indicates the amorphous feature of carbon in Sb-NCF.

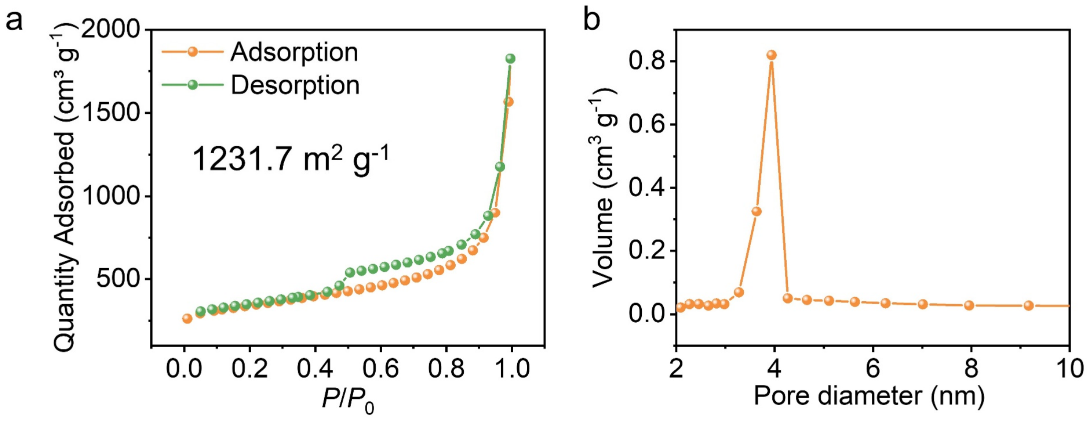

Supplementary Figure 18. a, N_2 adsorption-desorption isotherms of Sb-NCF. **b**, Pore size distribution of Sb-NCF. The adsorption-desorption isotherms of Sb-NCF exhibited a typical type-II hysteresis loop at the relative pressure between 0.45 and 0.99, suggesting the presence of abundant mesopores. The BET surface area for Sb-NCF was determined to be 1231.7 m^2 g^{-1}, which is larger than that of Sb-NSCF (445.4 m^2 g^{-1}). This could be related to the higher calcination temperature used for preparing Sb-NCF that resulted in more defective sites and porous structures in the carbon substrate.

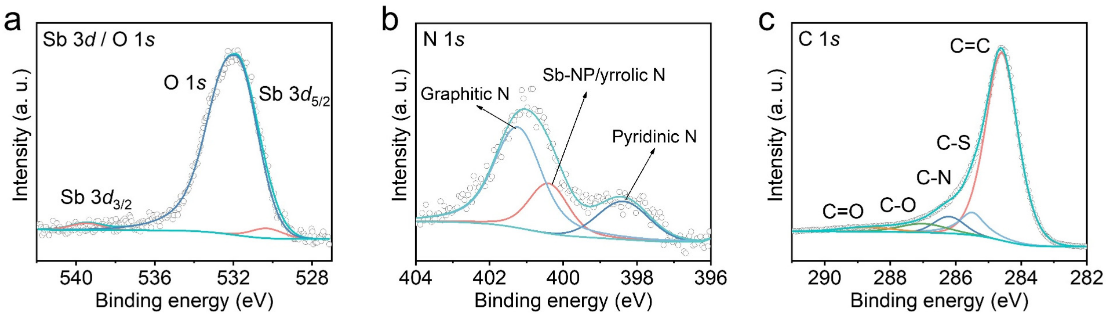

Supplementary Figure 19. a–c, high-resolution Sb 3*d* / O 1*s*, N 1*s* and C 1*s* spectrum of Sb-NCF. The high-resolution N 1*s* spectrum can be deconvoluted into three main peaks corresponding to pyridinic N (398.4 eV), Sb-N/pyrrolic N (400.4 eV) and graphitic N (401.2 eV), respectively.

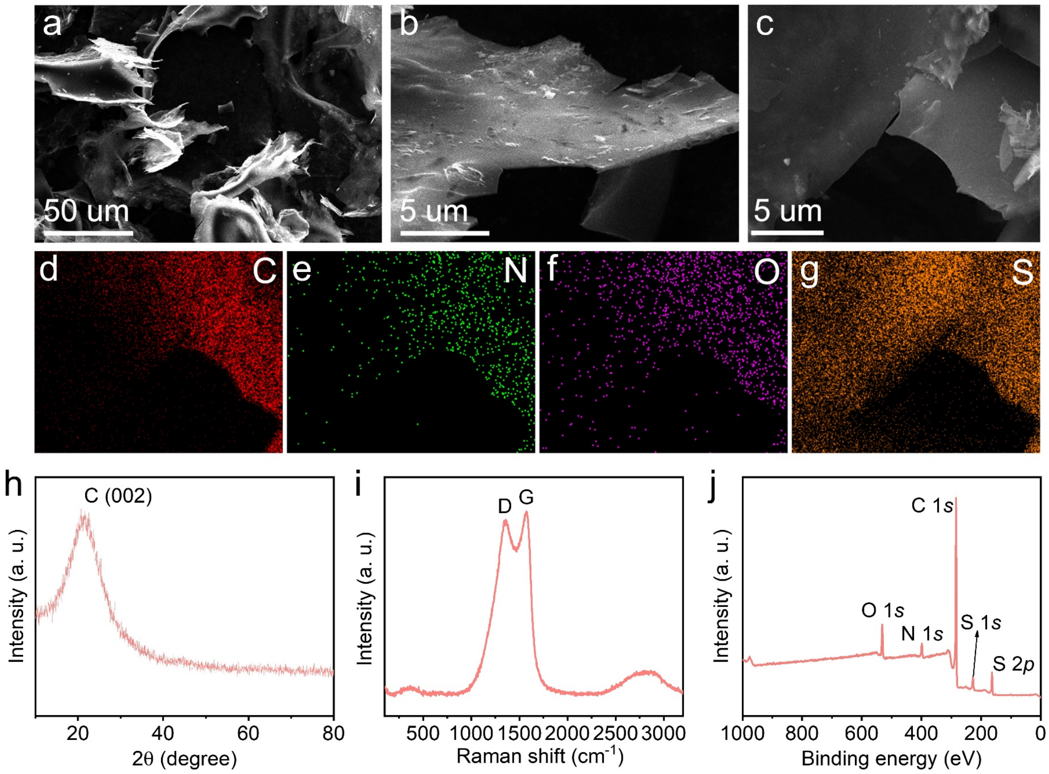

Supplementary Figure 20. a–c, SEM images of NSC at different magnifications. **d–g**, EDS elemental mapping of **c**, indicating the uniform distribution of C, N, O, and S elements in NSC. **h–j**, XRD pattern, Raman spectrum, and XPS survey spectrum of NSC. XRD pattern shows only a peak corresponding to C (002). Raman spectrum shows the amorphous carbon structure in NSC. XPS survey spectrum suggests that NSC is composed of C, N, O, S elements.

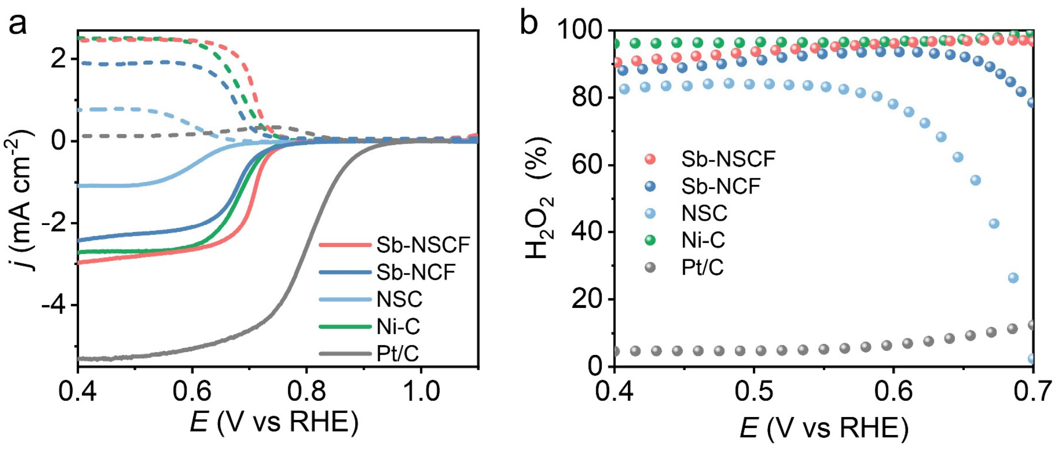

Supplementary Figure 21. a, Electrochemical oxygen reduction polarization curves (solid lines) at a rotation of 1600 rpm and simultaneous H_2O_2 detection currents on the ring electrode (dashed lines) for Sb-NSCF, Sb-NCF, NSC, Ni-C and Pt/C in O_2-saturated 0.1 M KOH electrolyte. **b**, Calculated H_2O_2 selectivity (%) on Sb-NSCF, Sb-NCF, NSC, Ni-C and Pt/C based on the RRDE measurements. Two reference samples were used: one was commercial Pt/C and the other was nickel nanoparticles supported on carbon black prepared (denoted as Ni-C) according to the reference[5]. The results show that Ni-C preferred the 2-electron ORR, while Pt/C favored the 4-electron ORR.

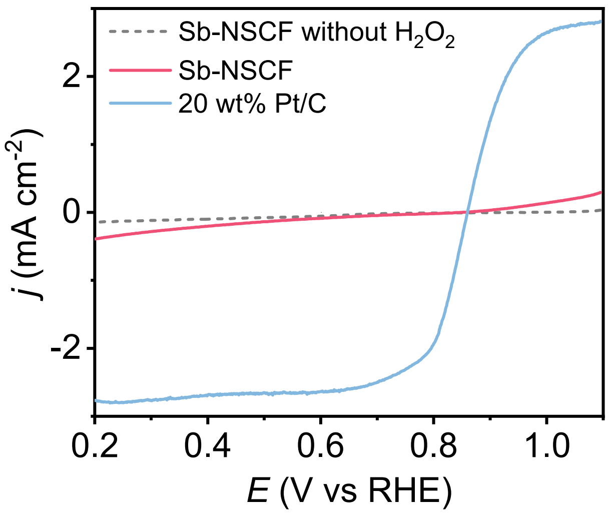

Supplementary Figure 22. Peroxide reduction reaction for Sb-NSCF and 20 wt% Pt/C in N_2-saturated 0.1 M KOH electrolyte with or without 3.5 mM H_2O_2.

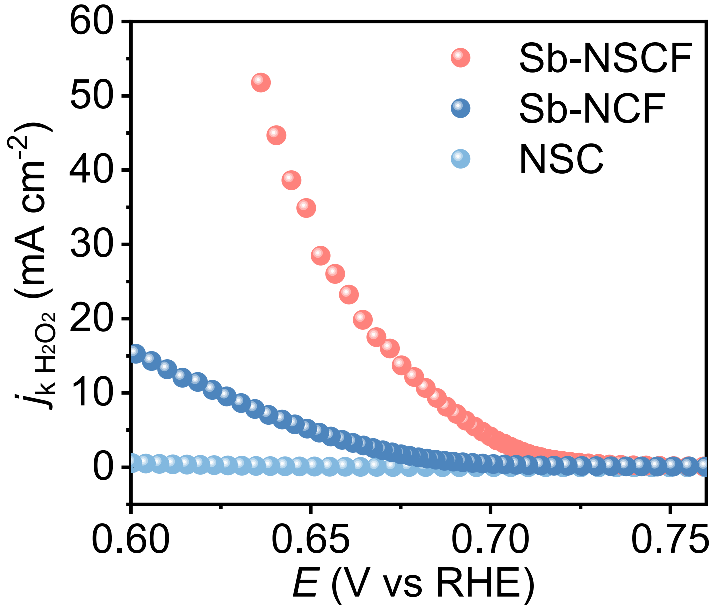

Supplementary Figure 23. Comparison of j_k at different potentials for different samples. The results show that Sb-NSCF possesses much larger values of j_k at a wide range of potential than Sb-NCF and NSC, manifesting its outstanding ORR kinetics for H_2O_2 production.

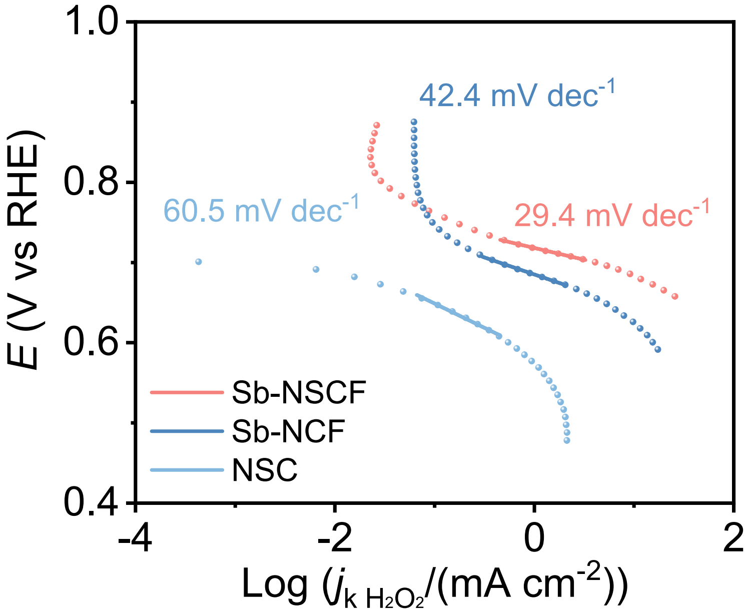

Supplementary Figure 24. Tafel slopes for different samples.

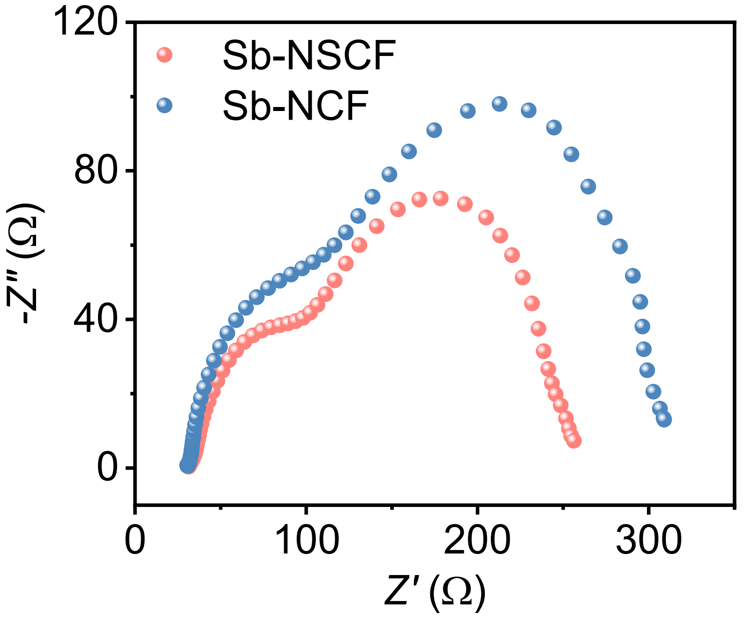

Supplementary Figure 25. Nyquist plots of Sb-NSCF and Sb-NCF from EIS measured at 0.616 V vs RHE. The results show that Sb-NSCF possesses a much smaller semicircle diameter than Sb-NCF, indicating the faster charge transfer rate for Sb-NSCF in the ORR process.

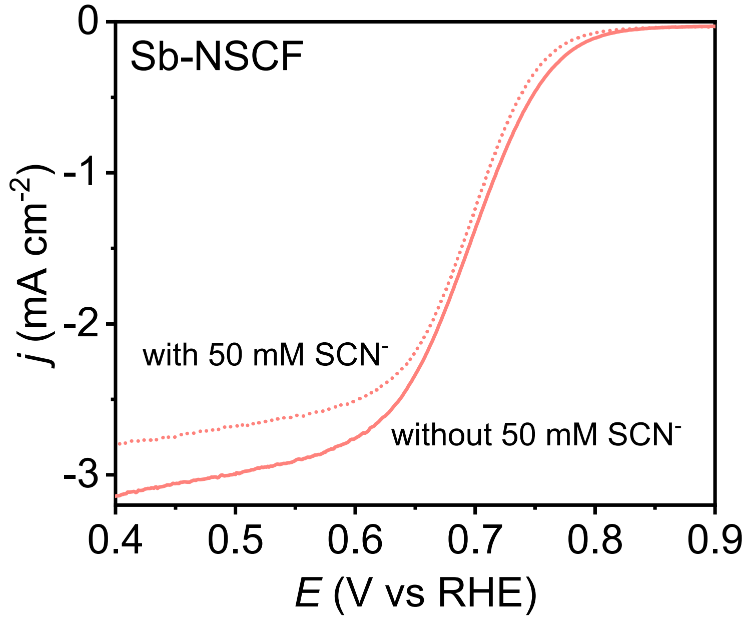

Supplementary Figure 26. ORR polarization curves of Sb-NSCF before and after poisoned by 50 mM KSCN. Prior to the measurement of LSV, the electrodes were dipped in 0.1 M KOH containing 50 mM KSCN for 4 h to fully poison the Sb active sites[6].

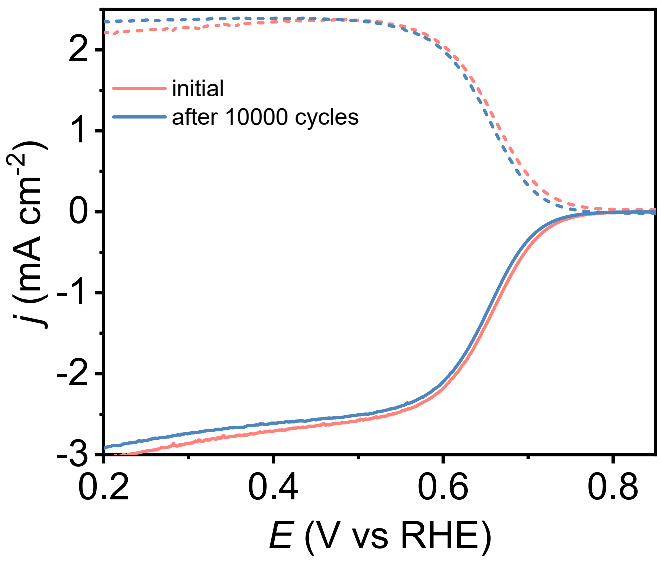

Supplementary Figure 27. Comparison of RRDE voltammograms of Sb-NSCF before and after 10000 cycles CV test.

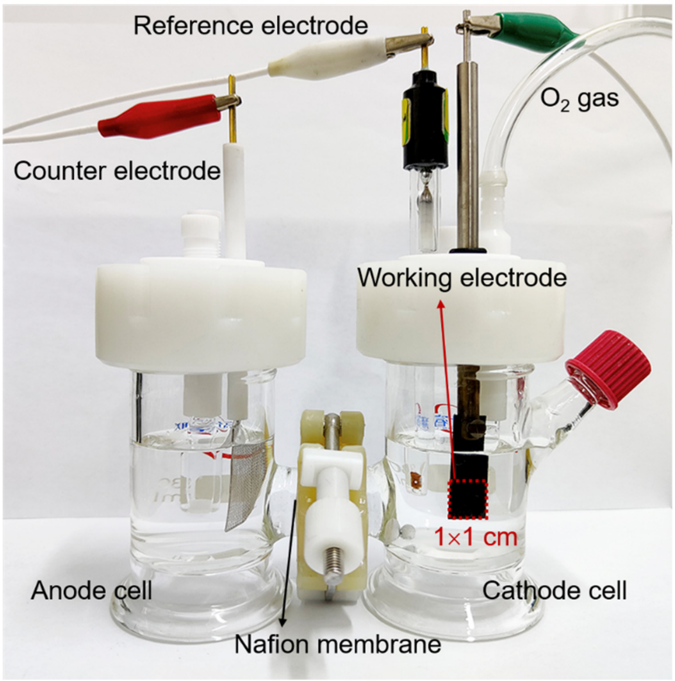

Supplementary Figure 28. Digital photograph of the three-electrode two-compartment H-cell for the bulk electrocatalytic production of H_2O_2.

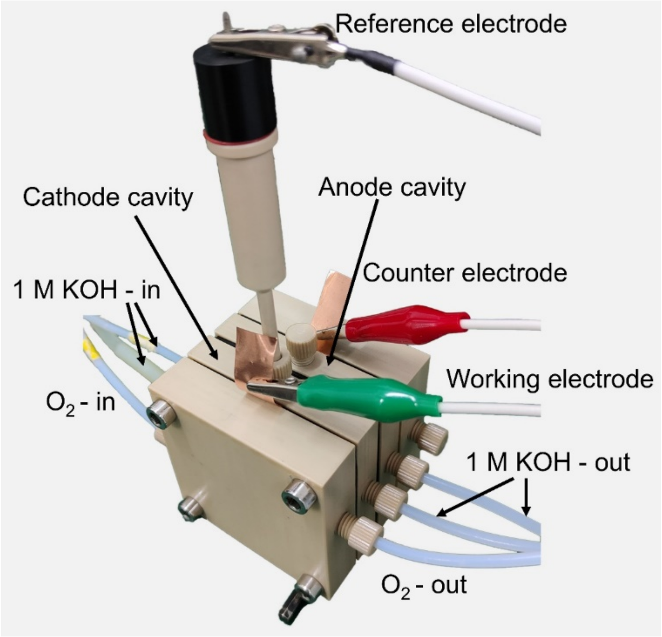

Supplementary Figure 29. Digital photograph of a flow cell setup for the electrochemical 2e⁻ ORR measurements.

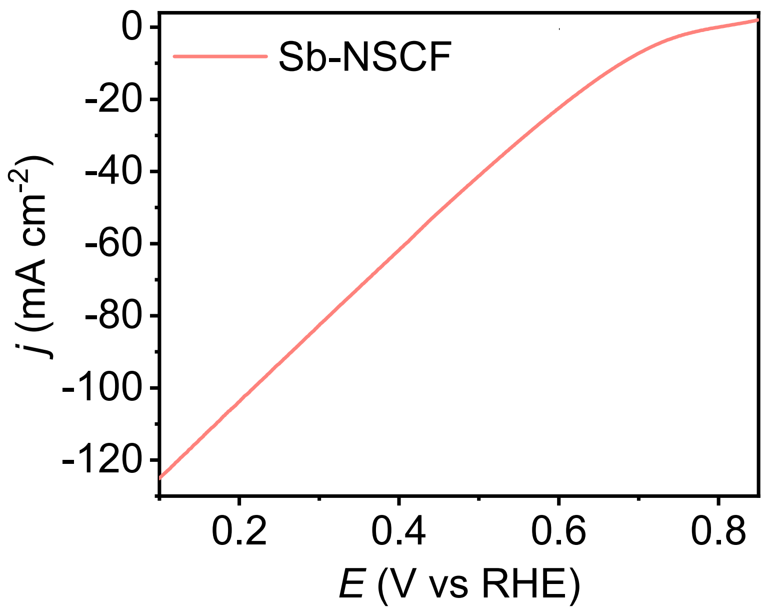

Supplementary Figure 30. LSV of Sb-NSCF measured by a three-phase flow cell setup with a gas diffusion electrode (GDE).

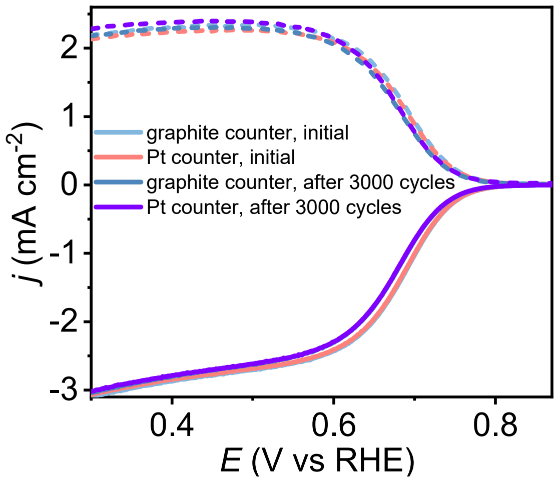

Supplementary Figure 31. Electrochemical oxygen reduction polarization curves (solid lines) at a rotation of 1600 rpm and simultaneous H_2O_2 detection currents on the ring electrode (dashed lines) for Sb-NSCF in O_2-saturated 0.1 M KOH electrolyte using Pt and graphite counter electrodes. The results show that the RRDE polarization curves before and after 3000 cycles obtained with a graphite counter electrode almost overlapped with those with a Pt counter electrode, indicating that the activity, selectivity and stability of the catalyst was not influenced by the Pt counter electrode.

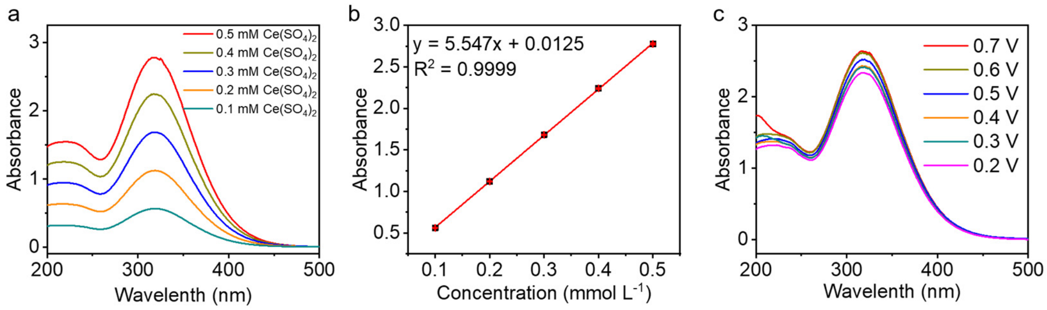

Supplementary Figure 32. a, Absorption spectra of a series of $Ce(SO_4)_2$ solutions with known concentration conducted on ultraviolet-visible spectrophotometer (GENESYS 180) at the range of 200 – 500 nm. **b**, Linear calibration curve based on the peak absorbance at 317 nm. **c**, Absorption spectra of $Ce(SO_4)_2$ solution with electrolytes electrolyzed at different potentials with 10 min. To determine the yield of H_2O_2, 100 μL of electrolyte at different potentials after neutralization was added into 3.0 mL of 0.5 mM $Ce(SO_4)_2$ solution.

Supplementary Tables

Supplementary Table 1. Comparison of metal weight content in Sb-NSCF with other reported main-group metal-based single atom catalysts.

Catalyst	Metal content (wt%)	Ref.
Sb-NSCF	10.32-XPS / 10.1-TG	This work
In SAs/NSBC	0.97-ICP	*Angew. Chem. Int. Ed.* **61**, e202117347 (2022)
Se-NCM	3.23-XPS	*Angew. Chem. Int. Ed.* **58**, 13466-13471 (2019)
Se-CNs	1.31-ICP	*Adv. Funct. Mater.* **30**, 1906194 (2020)
Sb SAC	0.79-ICP	*Angew. Chem. Int. Ed.* **60**, 21237-21241 (2021)
Mg-C_3N_4	0.401-ICP	*Angew. Chem. Int. Ed.* **60**, 25241-25245 (2021)
Sn-NC	3.78-ICP	*Nat. Mater.* **19**, 1215-1223 (2020)
Ca-N, O/C	1.19-ICP	*Adv. Mater.* **33**, 2107103 (2021)
Sb SAs/NC	2.86-ICP	*Energy Environ. Sci.* **13**, 2856-2863 (2020)
Se@NC	2.09-ICP	*Angew. Chem. Int. Ed.* **61**, e202114441 (2022)
Sb_1/NG(O)	2.3-ICP	*Angew. Chem. Int. Ed.* **61**, e202202200 (2022)
Sb-SAPC15	10.9-ICP	*Nat. Catal.* **4**, 374-384 (2021)
Bi SAs/C	2.13-ICP	*ChemSusChem* **13**, 6307-6311 (2020)
Sn@HCN	10.2-TG	*Sci. Adv.* **8**, eabm7489 (2022)
In_A/NC	0.4-ICP	*J. Am. Chem. Soc.* **143**, 6877-6885 (2021)
Mg-N-C	1.32-ICP	*Nat. Commun.* **11**, 938 (2020)
Sb-N-C	1.36-ICP	*Chem. Eng. J.* **439**, 135700 (2022)
In@NC-1000	0.57-ICP	*ACS Catal,* **12**, 7386-7395 (2022).

Supplementary Table 2. Fitting parameters[a] of Sb K-edge EXAFS curves.

Sample	Path	*N*	*R* (Å)	σ^2 (Å^2)	ΔE_0 (eV)	*R* factor
Sb-NSCF [b]	Sb-N	3.9	1.93	0.006	-8.5	0.0005
	Sb-C	3.3	2.64	0.005	-2.1	

[a] *N*, coordination number; *R*, distance between absorber and backscatter atoms; σ^2, Debye-Waller factor to account for both thermal and structural disorders; ΔE_0, inner potential correction; *R*-factor indicates the goodness of the fit. Error bounds (accuracies) that characterize the structural parameters obtained by EXAFS spectroscopy were estimated as $N \pm 20\%$; $R \pm 1\%$; $\sigma^2 \pm 20\%$; $\Delta E_0 \pm 20\%$. S_0^2 was fixed to 0.90. [b] Fitting range: $2.5 \leq k$ (/Å) ≤ 10.0 and $1.0 \leq R$ (Å) ≤ 2.3.

Supplementary Table 3. Comparison of the catalytic performance of Sb-NSCF with previously reported catalysts.

Catalyst	Electrolyte (0.1 M)	Onset potential (V)	Tafel (mV dec^{-1})	Mass activity ($A\ g^{-1}$) at 0.65 V	Productivity ($mmol\ g^{-1}_{cat}\ h^{-1}$)	Max. selectivity (%)	reference
Sb-NSCF	KOH	0.76	29.4	114.9	7460	97.2	This work
Co-N_2-C/HO	KOH	0.801	51.3	44.4	6912	91.3	*Adv. Funct. Mater.* **32**, 2106886 (2022)
Co-N-C	KOH	0.811	50	12.1	193.1	~66	*J. Am. Chem. Soc.* **141**, 12372-12381 (2019)
Ni-N_2O_2/C	KOH	0.66	/	1.10	46.2	96	*Angew. Chem. Int. Ed.* **59**, 13057-13062 (2020)
O-C(Al)	KOH	0.815	52	28.5	510	97	*Nat. Commun.* **11**, 5478 (2020)
Mo/-OSGH	KOH	0.775	54.7	20.86	/	95.8	*Angew. Chem. Int. Ed.* **59**, 9171-9176 (2020)
F-mrGO	KOH	0.76	/	144.8	/	~100	*Nat. Catal.* **1**, 282-290 (2018)
O-CNTs	KOH	0.77	47	20.6	111.7	~90	*Nat. Catal.* **1**, 156-162 (2018)
Co-NG(O)	KOH	0.79	/	155	418	80	*Nat. Mater.* **19**, 436-442 (2020)
Co-POC-O	KOH	0.83	34	16.5	/	85.6	*Adv. Mater.* **31**, 1808173 (2019)
Fe-CNT	KOH	0.815	/	23.9	677.7	95	*Nat. Commun.* **10**, 3997 (2019)
Ni-SA/G	KOH	0.74	60	/	/	/	*ACS Appl. Mater. Interfaces* **12**, 17519-17527 (2020)
$GNP_{C=O,1}$	KOH	0.815	48	8.87	/	97.7	*Nat. Commun.* **11**, 2209 (2020)
N-FLG-8	KOH	0.761	/	14.65	/	99.2	*Adv. Energy Mater.* **10**, 2000789 (2020)
AC-CO2B	KOH	~0.82	73	/	/	82	*Appl. Catal., B* **286**, 119860 (2021)
OCNS	KOH	~0.795	43	14.5	770	90	*Angew. Chem. Int. Ed.* **60**, 16607-16614 (2021)
NT-3DFG	KOH	~0.78	54.8	/	/	94	*ACS Catal.* **10**, 1993-2008 (2020)
GOMC	KOH	~0.78	48	/	/	93	*Angew. Chem. Int. Ed.* **58**, 1100-1105 (2019)
In SAs/NSBC	KOH	~0.78	30.3	2.5	/	94	*Angew. Chem. Int. Ed.* **61**, e202117347 (2022)
W/NO-C	KOH	0.815	115	/	/	97	*Adv. Funct. Mater.* **32**, 2110224 (2022)
CoN@CNTs	$HClO_4$	0.63	110	0.35	/	>99	*Nat. Commun.* **11**, 4181 (2020)
Co-NOC	$HClO_4$	~0.55	70	25.6	590	/	*J. Am. Chem. Soc.* **143**, 7819-7827 (2021)
Co-NC	$HClO_4$	0.672	110	9.04	275	81	*Chem* **6**, 658-674 (2020)
Pdδ+-OCNT	$HClO_4$	0.581	/	1.27	1700	98	*Nat. Commun.* **11**, 2178 (2020)
h-Pt-CuSx	$HClO_4$	~0.55	/	~8.25	546	96	*Chem* **5**, 2099-2110 (2019)

Supplementary References

1. Jing W. T., Zhang Y., Gu Y., Zhu Y. F., Yang C. C., Jiang Q. N-doped carbon nanonecklaces with encapsulated Sb as a sodium-ion battery anode. *Matter* **1**, 720-733 (2019).
2. Wang S., et al. Multifunctional sulfur-mediated strategy enabling fast-charging Sb_2S_3 micro-package anode for lithium-ion storage. *J. Mater. Chem. A* **9**, 7838-7847 (2021).
3. McCrory C. C. L., Jung S., Peters J. C., Jaramillo T. F. Benchmarking heterogeneous electrocatalysts for the oxygen evolution reaction. *J. Am. Chem. Soc.* **135**, 16977-16987 (2013).
4. Yan X., et al. Coupling highly dispersed Sb_2S_3 nanodots with nitrogen/sulfur dual-doped porous carbon nanosheets for efficient immobilization and catalysis of polysulfides conversion. *Chem. Eng. J.* **420**, 127688 (2021).
5. Song X., et al. Graphene-supported single nickel atom catalyst for highly selective and efficient hydrogen peroxide production. *ACS Appl. Mater. Interfaces* **12**, 17519-17527 (2020).
6. Zhang E., et al. Engineering the local atomic environments of indium single-atom catalysts for efficient electrochemical production of hydrogen peroxide. *Angew. Chem. Int. Ed.* **61**, e202117347 (2022).
